# Biocatalytic oligomerization-induced self-assembly of crystalline cellulose oligomers into nanoribbon networks assisted by organic solvents

**DOI:** 10.3762/bjnano.10.173

**Published:** 2019-08-26

**Authors:** Yuuki Hata, Yuka Fukaya, Toshiki Sawada, Masahito Nishiura, Takeshi Serizawa

**Affiliations:** 1 Department of Chemical Science and Engineering, School of Materials and Chemical Technology, Tokyo Institute of Technology, 2-12-1 Ookayama, Meguro-ku, Tokyo 152-8550, Japan; 2 Precursory Research for Embryonic Science and Technology (PRESTO), Japan Science and Technology Agency (JST), 4-1-8 Honcho, Kawaguchi-shi, Saitama 332-0012, Japan; 3 DKS Co. Ltd., 5 Ogawaracho, Kisshoin, Minami-ku, Kyoto-shi, Kyoto 601-8391, Japan

**Keywords:** cellulose oligomer, gel, nanoarchitectonics, nanoribbon networks, oligomerization-induced self-assembly, organic solvent

## Abstract

Crystalline poly- and oligosaccharides such as cellulose can form extremely robust assemblies, whereas the construction of self-assembled materials from such molecules is generally difficult due to their complicated chemical synthesis and low solubility in solvents. Enzyme-catalyzed oligomerization-induced self-assembly has been shown to be promising for creating nanoarchitectured crystalline oligosaccharide materials. However, the controlled self-assembly into organized hierarchical structures based on a simple method is still challenging. Herein, we demonstrate that the use of organic solvents as small-molecule additives allows for control of the oligomerization-induced self-assembly of cellulose oligomers into hierarchical nanoribbon network structures. In this study, we dealt with the cellodextrin phosphorylase-catalyzed oligomerization of phosphorylated glucose monomers from ᴅ-glucose primers, which produce precipitates of nanosheet-shaped crystals in aqueous solution. The addition of appropriate organic solvents to the oligomerization system was found to result in well-grown nanoribbon networks. The organic solvents appeared to prevent irregular aggregation and subsequent precipitation of the nanosheets via solvation for further growth into the well-grown higher-order structures. This finding indicates that small-molecule additives provide control over the self-assembly of crystalline oligosaccharides for the creation of hierarchically structured materials with high robustness in a simple manner.

## Introduction

Nanoarchitectonics is an emerging concept based on nanotechnology and other scientific fields, such as supramolecular chemistry, for constructing functional materials and systems in a bottom-up manner with the harmonization of mutual interactions [1–11]. Such harmonized mechanisms are found ubiquitously in biological systems consisting of a huge number of components; biomolecules, such as DNAs and peptides, and even living cells have therefore attracted considerable attention in nanoarchitectonics [1,4,11]. Achievements include nanopatterning [12], drug delivery [13], molecular sensing [14], nanodevices [15–16], and cell architectures [17–18]. On the other hand, crystalline poly- and oligosaccharides, such as cellulose and chitin, lag behind in nanoarchitectonics despite the superiority of their assemblies in terms of physicochemical stability and mechanical properties [19–20]. Plausible reasons are their complicated chemical synthesis [21] and low solubility in solvents [22–23], which prevent crystalline poly- and oligosaccharides from undergoing controlled self-assembly into ordered nanostructures in vitro. Nevertheless, naturally derived nanostructures (called nanocellulose [19,24–25] and nanochitin [20,26]) have demonstrated a robustness that makes them attractive for a wide range of applications. Therefore, the use of crystalline poly- and oligosaccharides as molecular building blocks has the potential to open new horizons in nanoarchitectonics.

Oligomerization-induced self-assembly is a promising method for overcoming the above issues (i.e., the complicated chemical synthesis and the low solubility in solvents) with crystalline oligosaccharide nanoarchitectonics [21,27–28]. For example, the cellulase-catalyzed oligomerization of β-ᴅ-cellobiosyl fluoride monomers [29] and the cellodextrin phosphorylase (CDP)-catalyzed oligomerization of α-ᴅ-glucose 1-phosphate (αG1P) monomers from ᴅ-glucose [30–31] and cellobiose [32–33] primers have been demonstrated, where the synthesized cellulose oligomers (also known as cellodextrin) self-assemble in situ into unique nanostructures. In addition to the plain cellulose oligomer, cellulose oligomer derivatives bearing azido [34], alkyl [35], oligo(ethylene glycol) [36], vinyl [37–38], and amino [39–40] groups at the terminal have been successfully synthesized by using glucose derivatives as primers for the CDP-catalyzed oligomerization. By exploiting those enzyme-catalyzed oligomerization systems, various nanostructures, including nanofibrous assemblies [41], rectangular nanosheet-shaped lamellar crystals [30–31,39,42], distorted nanosheets with a bilayer structure [35], helical nanorods with a bilayer structure [35], and network structures composed of nanoribbon-shaped lamellar crystals [33,42–46] have been successfully constructed by changing the enzymatic reactions, tuning the self-assembly kinetics, introducing terminal functional groups, and using additives. Among them, the strategy using additives has the advantages of versatility and convenience. Polymers [43–44] and colloidal particles [45] were shown to be useful additives. However, the potential of small-molecule additives for controlling the oligomerization-induced self-assembly of cellulose oligomers has yet to be investigated systematically, even though many more candidates are available for small molecules than for polymers and colloidal particles.

**Figure 1:**
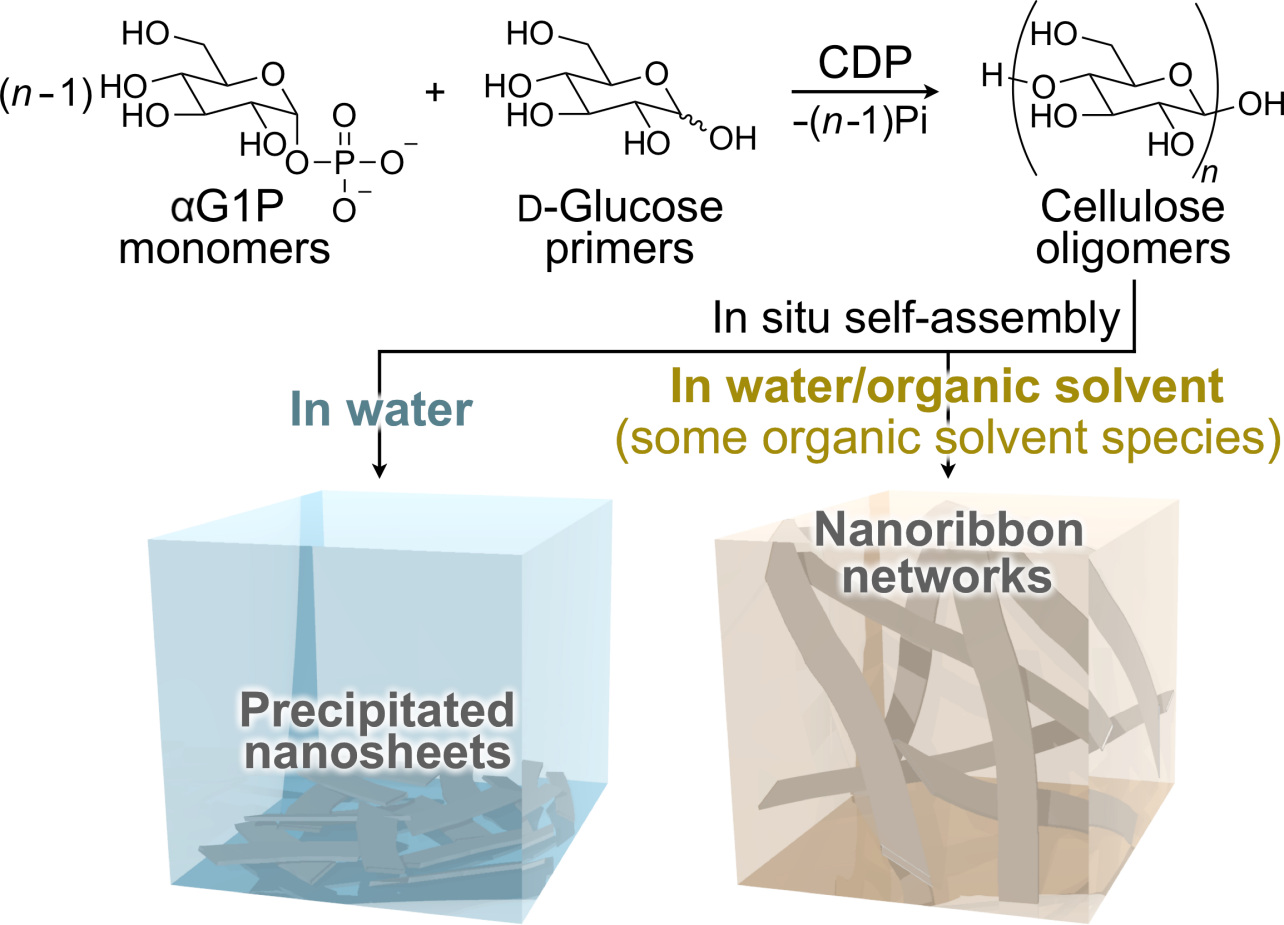
Reaction scheme of the CDP-catalyzed oligomerization and schematic illustrations of cellulose oligomer assemblies produced in aqueous buffer solutions and in mixtures of an aqueous buffer solution and appropriate organic solvents.

Herein, we show the formation of nanoribbon networks composed of crystalline cellulose oligomers via oligomerization-induced self-assembly assisted by organic solvents, which are widely used typical small molecules. The CDP-catalyzed oligomerization from ᴅ-glucose primers, which is known to produce rectangular nanosheets as precipitates in aqueous solution (Figure 1) [30–31], was used in this study. The oligomerization system in mixtures of an aqueous buffer solution and appropriate organic solvents was found to result in nanoribbon network structures for gel formation (Figure 1). It was suggested that the precipitation of the nanosheets was prevented effectively via solvation with the organic solvents through hydrogen bonding, allowing the formation of well-grown higher-order structures (i.e., nanoribbon networks). The observation demonstrates the significant effect of small-molecule additives for controlling the self-assembly of cellulose oligomers for the creation of hierarchically structured materials in a simple manner. This study will open a new scientific or technological world of nanostructured cellulose oligomers, which is different from that of naturally derived cellulosic materials [47–50].

## Results and Discussion

**Figure 2:**
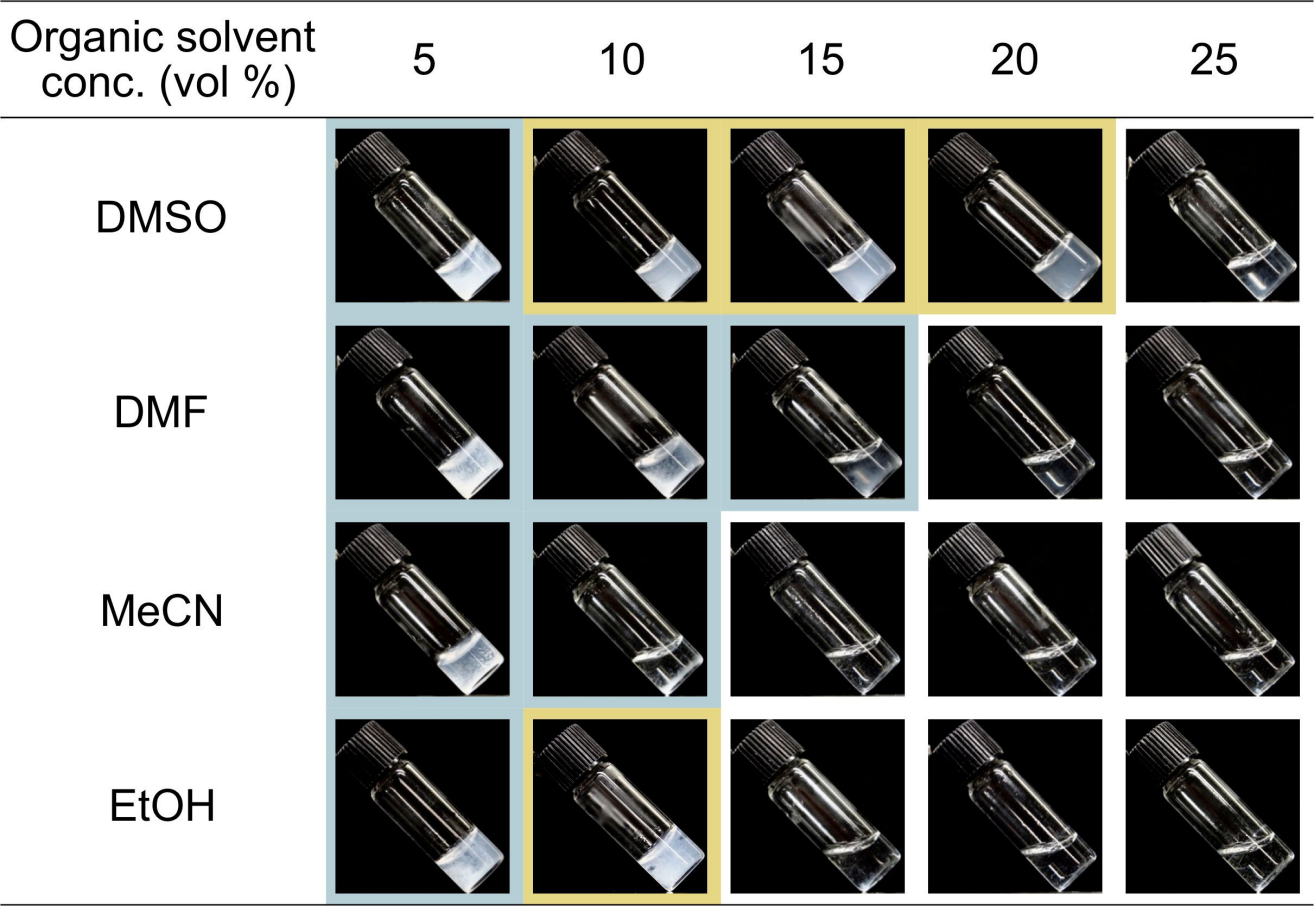
Photographs of the reaction mixtures with organic solvents after the CDP-catalyzed oligomerization reaction. The blue, yellow, and white backgrounds denote the precipitate state, gel state, and trace amount of the products, respectively.

Four kinds of common water-miscible organic solvents, namely, dimethyl sulfoxide (DMSO), *N*,*N*-dimethylformamide (DMF), acetonitrile (MeCN), and ethanol (EtOH), with different characteristics were used in this study. We addressed the CDP-catalyzed oligomerization from ᴅ-glucose primers, where the precipitated nanosheets are produced in aqueous solution [30–31]. The oligomerization reaction was conducted in the presence of organic solvents (5–25 vol %), while other conditions, such as αG1P monomer concentration (0.2 M), ᴅ-glucose primer concentration (0.05 M), CDP concentration (0.2 U mL^−1^), temperature (60 °C), and incubation time (72 h), were as described in previous reports [31,42]. After the reaction, colorless solid products were observed in the solutions with relatively low organic solvent concentrations, suggesting the successful synthesis of water-insoluble cellulose oligomers under those conditions (Figure 2). Remarkably, the reaction mixtures with 10–20 vol % DMSO and 10 vol % EtOH were found to be in gel states after the reaction (Figure 2, photographs with yellow background).

**Figure 3:**
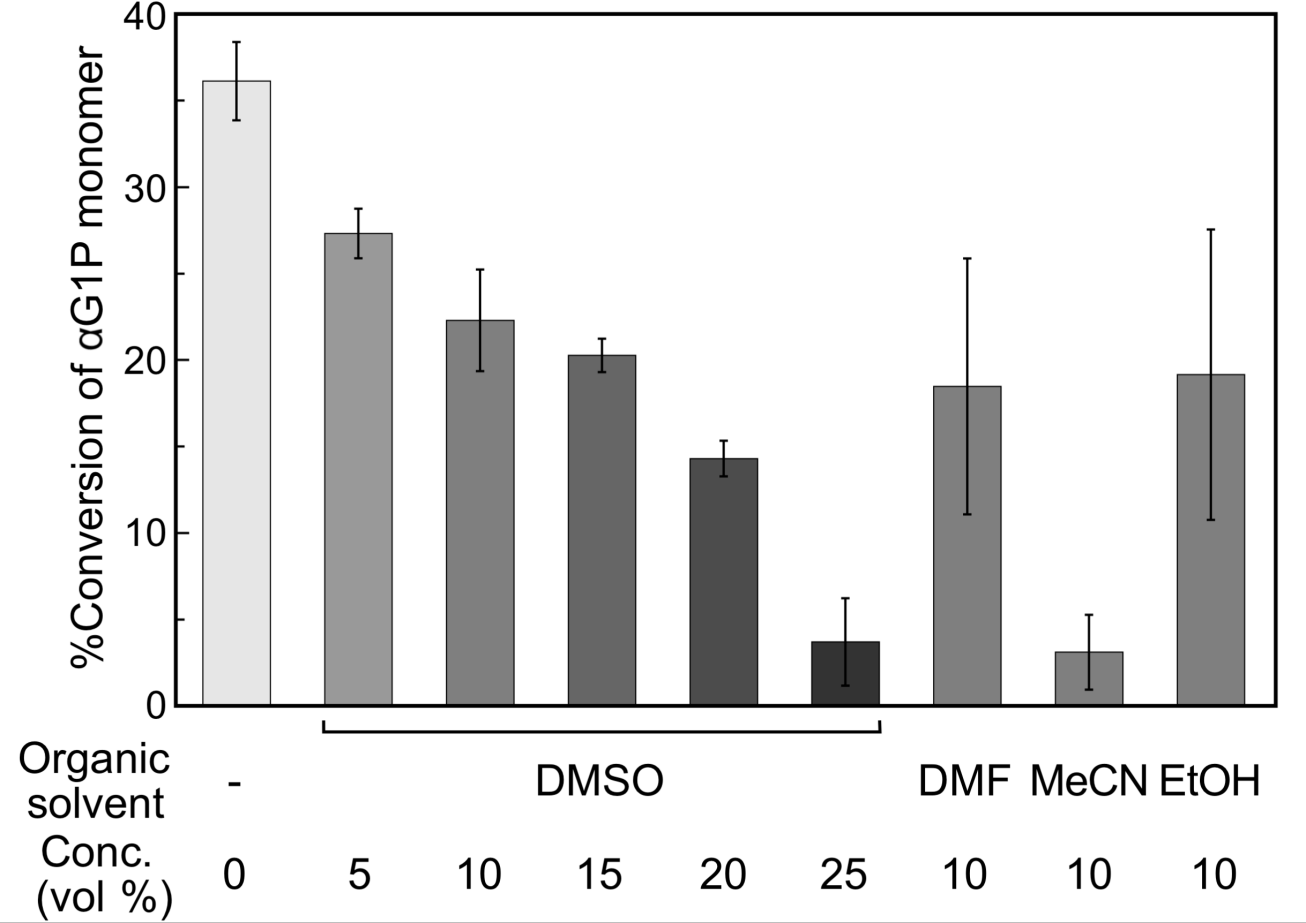
The αG1P monomer conversion into insoluble products with organic solvents.

**Figure 4:**
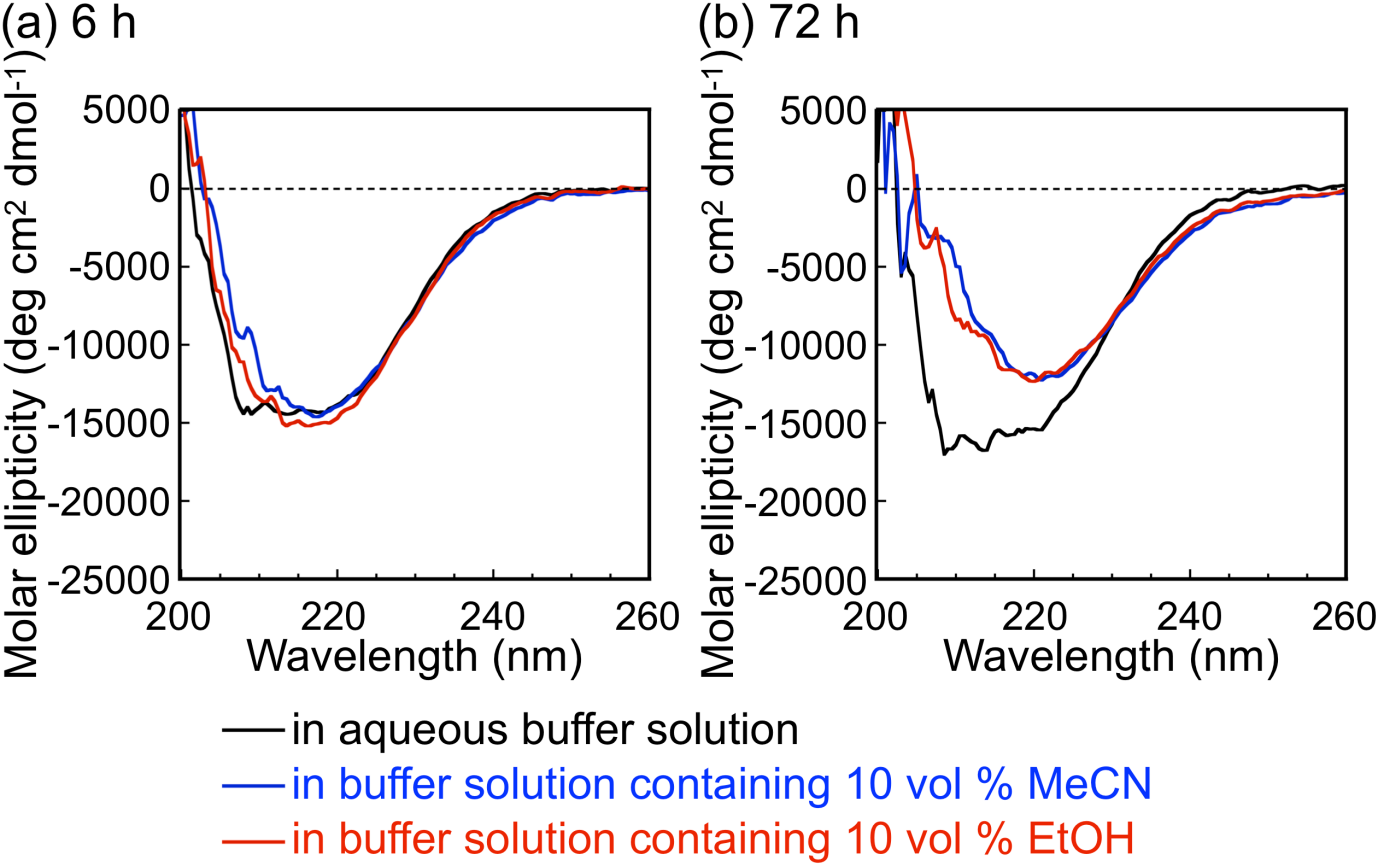
CD spectra of CDP in 8 mM phosphate buffer solution containing 10 vol % MeCN or EtOH after incubation at 60 °C for (a) 6 and (b) 72 h.

The apparent turbidity of the reaction mixtures decreased with increasing organic solvent concentrations for each organic solvent species (Figure 2). The observations were simply due to a reduction in the conversion of αG1P monomer into insoluble products (Figure 3), which was estimated from the insoluble product weights and the average degree of polymerization (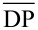) values calculated from the matrix-assisted laser desorption/ionization time-of-flight (MALDI–TOF) mass spectra (see below). To check the possible denaturation of CDP by the organic solvents as an explanation for the decreased monomer conversions, circular dichroism (CD) spectra of CDP solutions containing 10 vol % MeCN or EtOH were measured after incubation at 60 °C (Figure 4). Note that the light absorption of DMSO and DMF made the CD spectroscopy measurement impossible under the same conditions. Although incubation with the organic solvents for 6 h hardly affected the CD spectra (Figure 4a), incubation for 72 h led to a change in the spectra (Figure 4b), showing a change in the secondary structure of CDP. These results indicate that CDP was denatured gradually by the organic solvents during the oligomerization reaction, leading to decreasing enzymatic reaction rates for lower monomer conversions. On the other hand, although MeCN and EtOH caused different αG1P monomer conversions (Figure 3), they caused a similar change in the CD spectra of CDP. Each organic solvent species may lead to the denaturation of CDP in a different manner, while the difference could not be revealed by CD spectroscopy. Moreover, the organic solvents might affect the interaction between CDP and the substrates/products.

**Figure 5:**
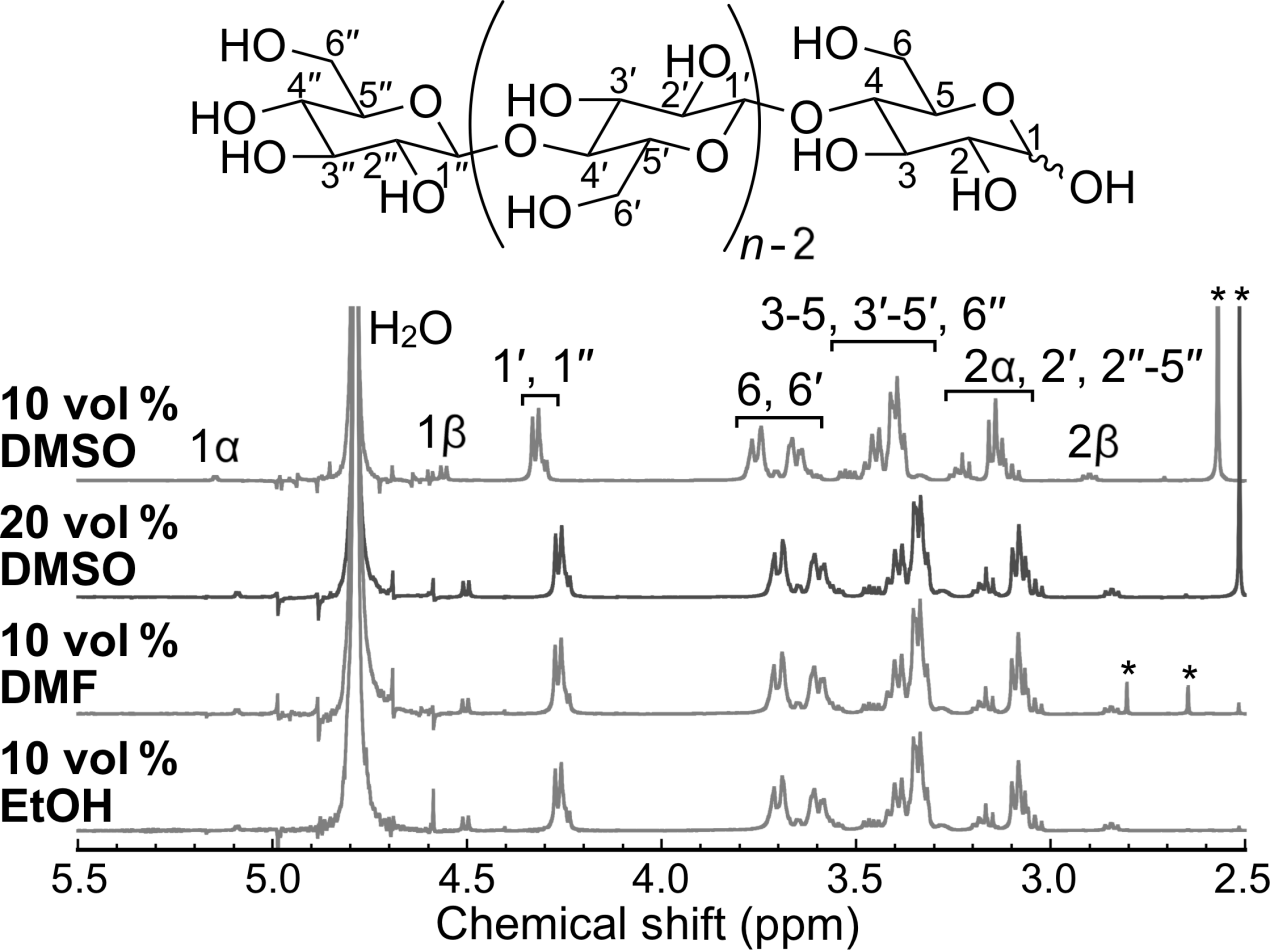
^1^H NMR spectra of the products with DMSO, DMF, and EtOH. The peaks with * are derived from the residual organic solvents.

**Figure 6:**
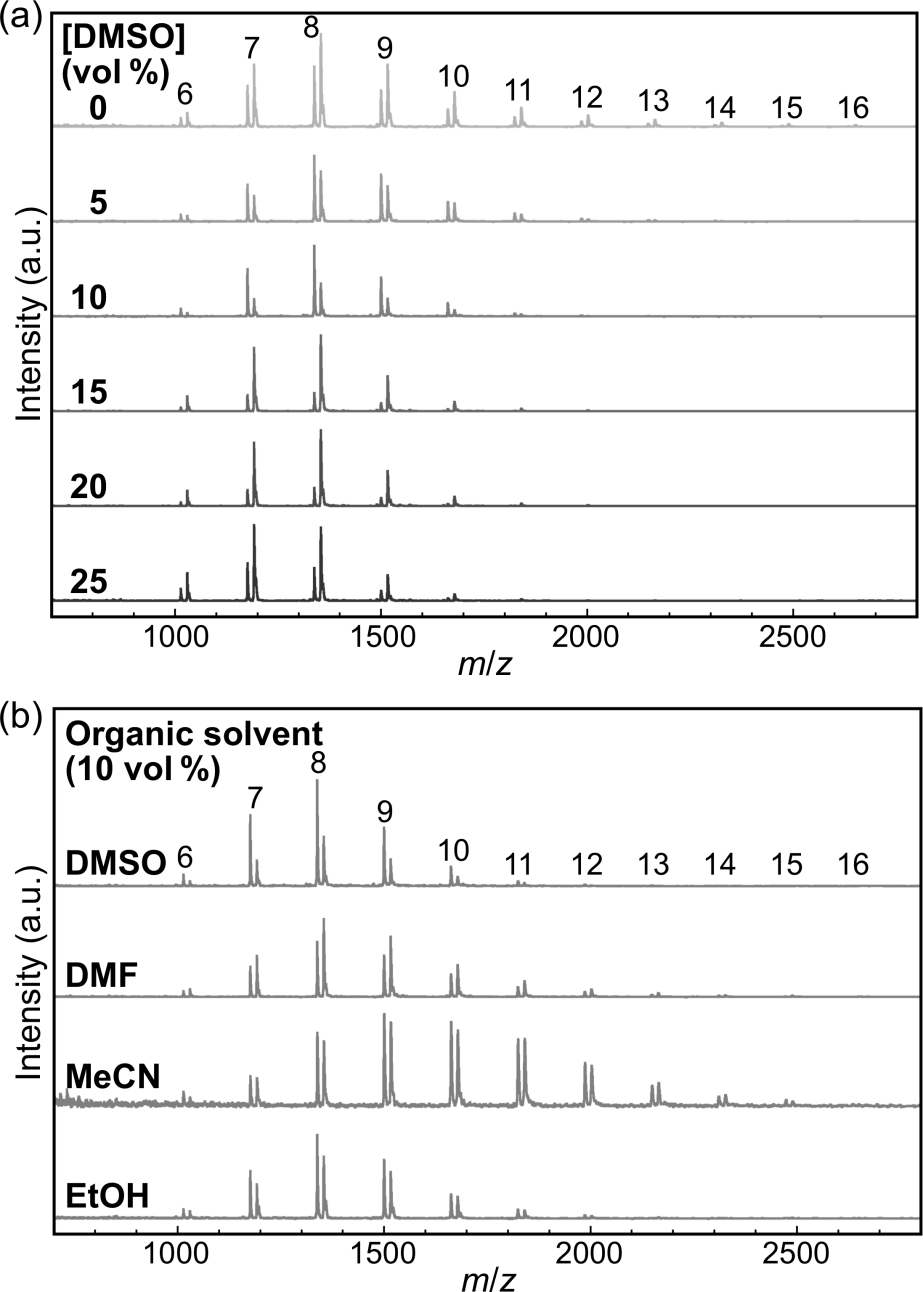
MALDI–TOF mass spectra of the products with (a) DMSO at various concentrations and (b) various organic solvents at 10 vol %. The numbers above the peaks denote the DP values of the cellulose oligomers. The spectra show two series of peaks corresponding to cellulose oligomers with sodium and potassium ion adducts.

The chemical structure of the products was analyzed by ^1^H NMR spectroscopy and MALDI–TOF mass spectrometry. The NMR spectra of the representative products showed proton signals for cellulose oligomers (Figure 5). In addition, the mass spectra further revealed the successful synthesis of cellulose oligomers (Figure 6). The 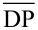 values were calculated from both kinds of spectra to be 8–10, slightly lower than that of the oligomers synthesized in aqueous solution (i.e., 10) [31,42], depending on the organic solvent species and their concentrations (Table 1). The slight decrease in 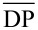 with the organic solvents was mainly attributed to the lower enzymatic reaction rates; a slower reaction would decrease the number of propagation steps for each molecular chain before solidification. Other factors, however, appeared to affect the 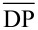 (e.g., 10 vol % MeCN caused a relatively low monomer conversion yet a relatively high 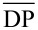, Figure 3 and Table 1). In addition, the population standard deviations (PSDs) of DP were calculated from the mass spectra and showed a trend of decreasing polydispersity with decreasing 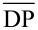 (Table 1), similar to the oligomerization in aqueous solution [42]. Although these results revealed a slight variation in the 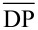 and polydispersity, our previous studies suggested that 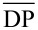 and polydispersity are not the dominant factors in the assembled structure of cellulose oligomers in the 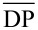 range of 7–10 [33,43–46]. Therefore, the gelation was considered not to be caused directly by the change in the DP, as discussed further below.

**Table 1:** Summary of CDP-catalyzed oligomerization reaction with organic solvents.

organic solvent		state of product	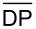		PSD of DP	allomorph	%χ_c_
species	concentration (vol %)	(inversion test)	(NMR)	(MALDI–TOF mass)	(MALDI–TOF mass)	(XRD and/or ATR-FTIR)	(XRD)

no organic solvent	0	precipitate [31,42]	10 [31,42]	9	1.9	cellulose II [31,42]	52 [42]
DMSO	5	precipitate	–	9	1.4	–	–
DMSO	10	gel	8	8	1.2	cellulose II	60
DMSO	15	gel	–	8	1.1	–	–
DMSO	20	gel	8	8	1.0	cellulose II	64
DMSO	25	trace amount of product	–	8	1.1	–	–
DMF	10	precipitate	9	9	1.6	cellulose II	–
MeCN	10	precipitate	–	10	1.9	–	–
EtOH	10	gel	9	9	1.3	cellulose II	62

**Figure 7:**
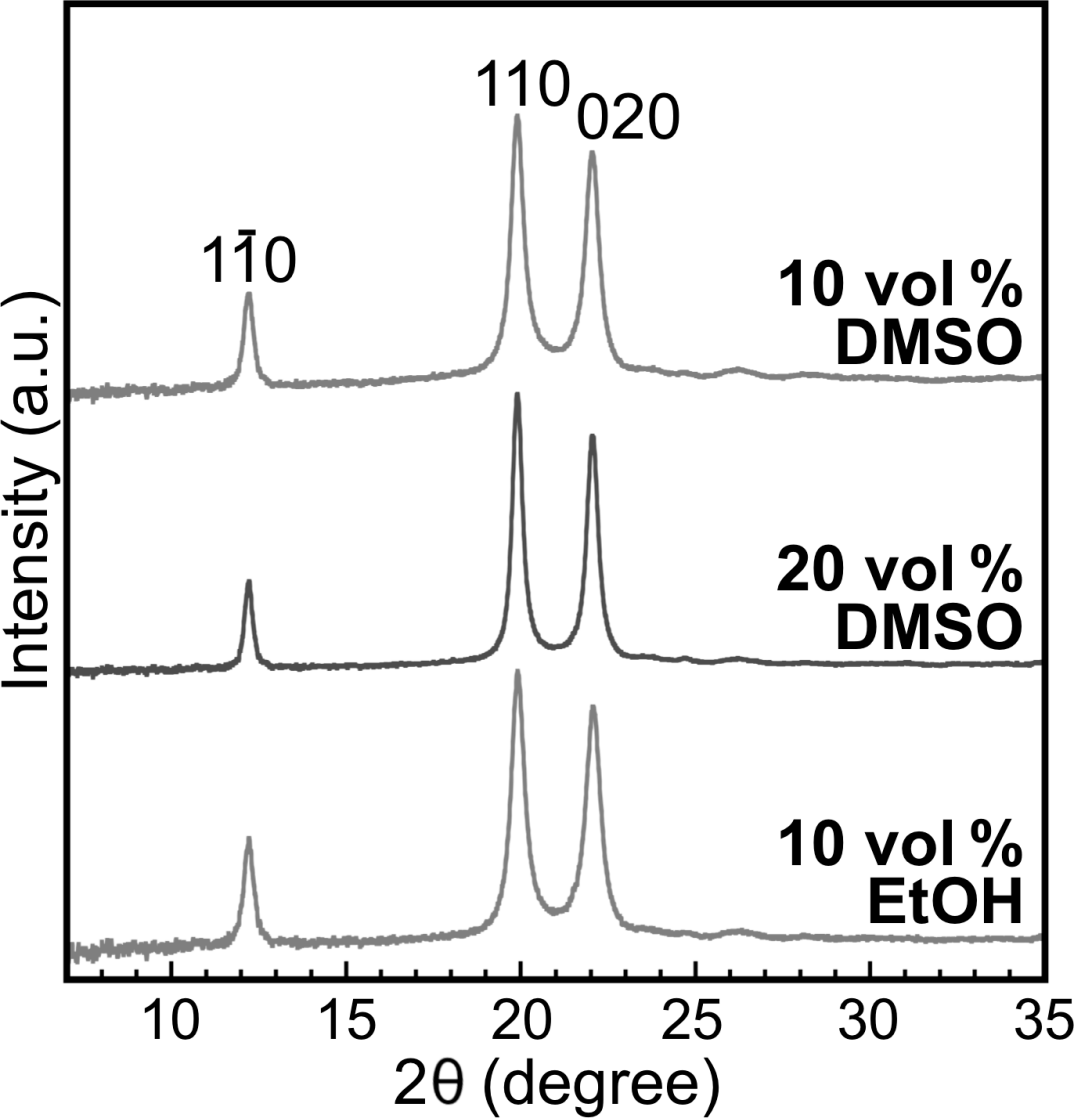
XRD profiles of the products with organic solvents. Miller indices for cellulose II are shown above the peaks.

**Figure 8:**
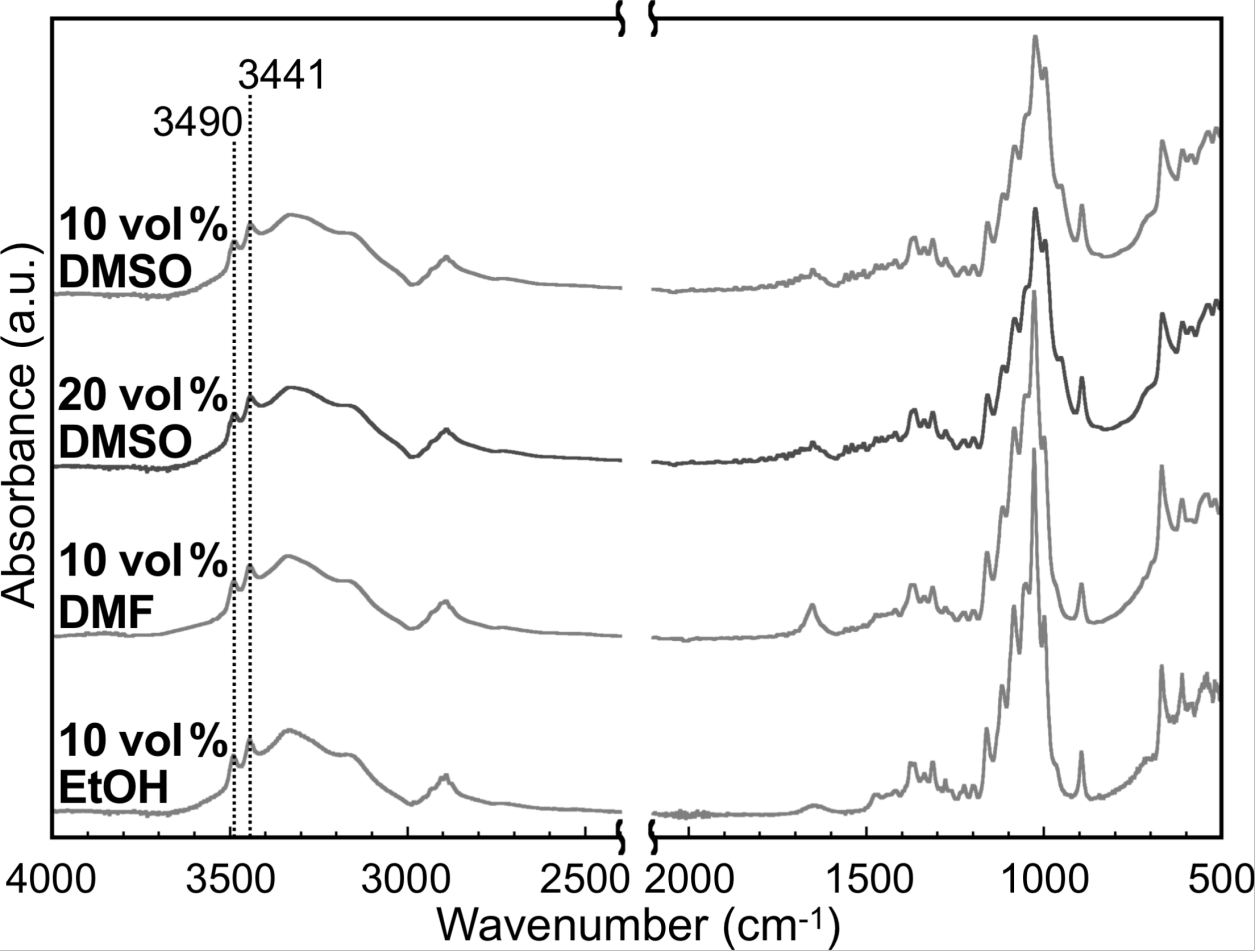
ATR-FTIR absorption spectra of the products with organic solvents. The numbers above the peaks denote the wavenumber.

The crystal structure of the representative products was analyzed by X-ray diffraction (XRD) measurements and attenuated total reflection Fourier-transform infrared (ATR-FTIR) absorption spectroscopy. The XRD profiles showed three peaks at 2θ (θ is the Bragg angle) of 12.2, 19.9, and 22.1° (Figure 7), which corresponded to 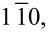 110, and 020 of the cellulose II allomorph, respectively [30]. In addition, the ATR-FTIR absorption spectra showed two characteristic peaks for the intrachain hydrogen-bonded hydroxyl groups in the cellulose II allomorph [51] at approximately 3441 and 3490 cm^−1^ (Figure 8). The cellulose II allomorph is the most stable allomorph of cellulose [19] and is typical of the cellulose oligomer assemblies formed in aqueous solution [31,42]. The degree of crystallinity (χ_c_) values of the gelled products were calculated from the XRD profiles and found to be higher than those of the products in aqueous solution [42] (Table 1). The higher crystallinity with the organic solvents was attributed to the lower polydispersity in the DP, which would decrease the amount of the amorphous-like assembled structures of the terminal residues of relatively long oligomer chains [42]. In other words, a higher uniformity of the chain lengths leads to higher integrity of the crystals.

**Figure 9:**
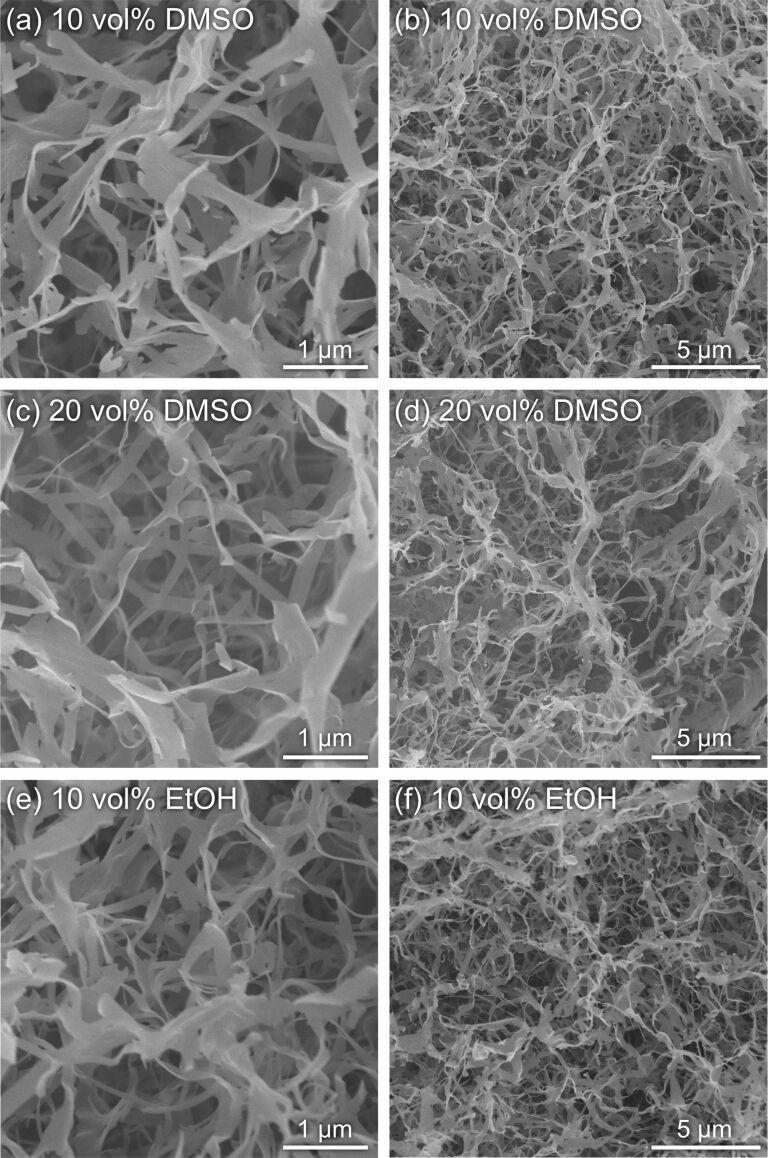
SEM images of the xerogels prepared from the gels synthesized with (a,b) 10 vol % DMSO, (c,d) 20 vol % DMSO, and (e,f) 10 vol % EtOH.

Scanning electron microscopy (SEM) was used to uncover the nanomorphology of the gels. The images revealed a well-grown network structure composed of nanoribbon-shaped fibers (Figure 9), which were similar in shape to lamellar crystals of cellulose oligomers [42,52–54]. According to our examination, the cross-linking of the nanoribbons was apparently based on their physical contact, possibly through the hydrophobic effect and hydrogen bonding. We previously demonstrated nanoribbon network formation via oligomerization-induced self-assembly under macromolecular crowding conditions [43–44], which represent a solution state with high macromolecular concentrations [55–57]. The crowding macromolecules with high molecular weights (typically more than 20k) induced high solution viscosity and depletion repulsion, which prevented the nanosheet-shaped lamellar crystals from aggregation and subsequent precipitation, enabling the formation of well-grown nanoribbon networks. On the other hand, the organic solvents used in this study were small molecules, indicating a different mechanism.

**Figure 10:**
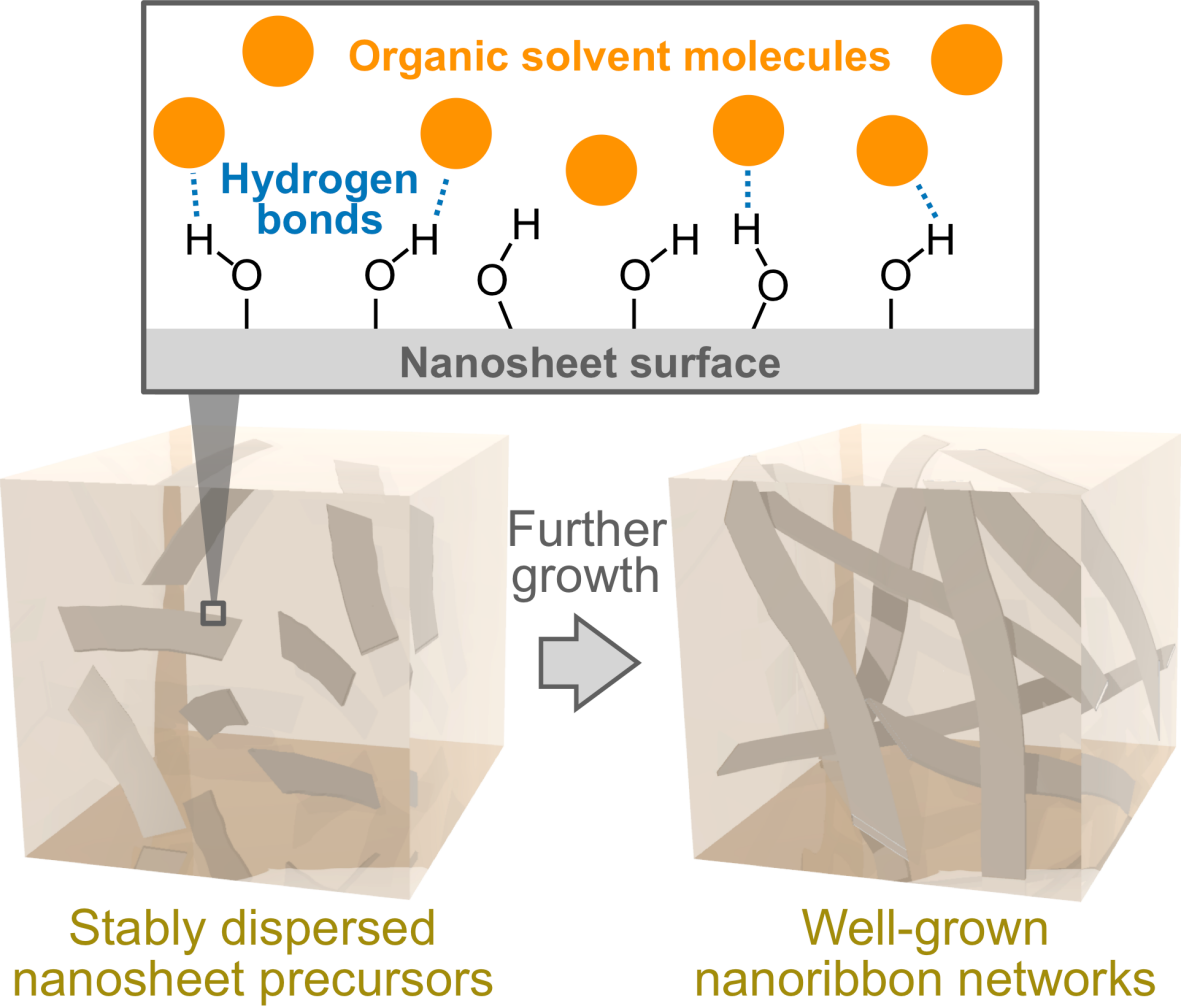
Schematic illustration of the proposed mechanism for dispersion stabilization of the nanosheet precursors via solvation with the organic solvents.

To gain insight into the mechanism underlying the nanoribbon network formation, we focused on the Kamlet–Taft solvent parameters, which are the most comprehensive and frequently used quantitative measure of solvent properties [58–59]. Among the three parameters, namely, the hydrogen bond donation ability (acidity) α, the hydrogen bond acceptor ability (basicity) β, and the dipolarity/polarizability π*, β was found to be correlated. The organic solvents with relatively high β-values were found to induce the nanoribbon network formation (Table 2). This finding suggests that the precipitation of the nanosheet precursors was prevented effectively via solvation with the organic solvents mainly through hydrogen bonding from hydroxyl hydrogen on cellulose oligomers to the organic solvents, allowing further growth into higher-order structures (i.e., nanoribbon networks) in the bulk solution (Figure 10). This proposed mechanism would be reasonable considering that, in the case of a cellulose solvent series, β is the most significant parameter for dissolving cellulose via a mechanism involving interaction through hydrogen bonding with cellulose [60]. Such an attractive interaction with additive molecules is a novel driving force for controlling the oligomerization-induced self-assembly of cellulose oligomers. In summary, it was shown that organic solvents had the potential to induce the formation of well-grown higher-order structures of crystalline cellulose oligomer assemblies.

**Table 2:** The Kamlet–Taft solvent parameters of the organic solvents used in this study [58].

Organic solvent	α	β	π*

DMSO	0	0.76	1.00
DMF	0	0.69	0.88
MeCN	0.19	0.40	0.75
EtOH	0.86	0.75	0.54

## Conclusion

We showed that organic solvents provided control over the oligomerization-induced self-assembly of cellulose oligomers. The organic solvents with relatively high β-values prevented the irregular aggregation of the particulate nanostructures for the formation of well-organized higher-order structures. The main driving force was suggested to be the interaction of the organic solvent molecules with cellulose oligomers. Therefore, the use of more strongly interacting molecules will allow more drastic changes in the assembled structures. Promising candidates include cellulose-dissolving solvents, represented by ionic liquids [23], which are known (or considered) to dissolve cellulose via direct interactions. Furthermore, the introduction of functional groups at the terminal of cellulose oligomers [34–40] significantly expands the variety of available molecular species that can interact with the oligomers. Consequently, the present study serves as inspiration for controlling the self-assembly of crystalline oligo- and polysaccharides via exploiting small-molecule additives, leading to advanced nanoarchitectonics for the creation of hierarchically structured materials with high robustness.

## Experimental

### Materials

αG1P disodium salt *n*-hydrate and 40% sodium deuteroxide (NaOD)/deuterium oxide (D_2_O) solution were purchased from Wako Pure Chemical Industries. ProteoMass MALDI–MS standard, 1% trifluoroacetic acid, MeCN used for preparing MALDI–TOF mass spectrometry samples, 2,5-dihydroxybenzoic acid, and D_2_O were purchased from Sigma-Aldrich. Dotite was purchased from Nisshin EM Corporation. All other reagents were purchased from Nacalai Tesque. Ultrapure water with a resistivity greater than 18.2 MΩ cm was supplied by a Milli-Q Advantage A-10 apparatus (Merck Millipore) and used throughout all experiments.

### CDP-catalyzed oligomerization reaction

CDP from *Clostridium thermocellum* YM4 was prepared using a genetically engineered *Escherichia coli* according to a previous report [31]. For the synthesis of cellulose oligomers with organic solvents, αG1P monomer (0.2 M) and ᴅ-glucose primer (0.05 M) were incubated with CDP (0.2 U mL^−1^) in 4-(2-hydroxyethyl)-1-piperazineethanesulfonic acid (HEPES; 0.5 M) buffer solutions containing organic solvents (DMSO, DMF, MeCN, or EtOH; 5, 10, 15, 20, or 25 vol %) at 60 °C for 72 h. Note that a HEPES buffer solution (1.5 M, pH 7.5) was used to prepare the reaction mixtures. To readily assess gelation, the vials containing the mixtures after the reaction were inverted. For SEM observations, the gelled products (1 mL) were purified by immersion in water at 4 °C for 1 week. The water was exchanged each day. For the other characterization techniques, the mixtures after the reaction (0.3 mL) were subjected to pipetting to obtain product dispersions. The resultant particulate products were purified with water/organic solvent mixtures (the organic solvent concentrations were the same as those in the reaction mixtures) by performing at least five centrifugation (20,400*g*)/redispersion cycles to remove more than 99.999% of the soluble fraction of the reaction mixtures. For MALDI–TOF mass spectrometry, the purified product dispersions were stored at 4 °C until use. For the quantification of the insoluble products, a volume of the purified product dispersions was dried at 105 °C for 24 h, followed by weighing. For ^1^H NMR spectroscopy, ATR-FTIR absorption spectroscopy, and XRD measurements, as much as possible of the supernatant after the final centrifugation was removed by pipette, followed by adding water to the products. The resultant product aqueous dispersions with residual organic solvents were lyophilized and then stored at 4 °C until use.

### Characterization of the products

For NMR spectroscopy, the lyophilized products were dissolved in 4% NaOD/D_2_O to obtain product solutions (≥2% (w/v)). ^1^H NMR spectra were recorded on an AVANCE III HD500 spectrometer (500 MHz, Bruker) at ambient temperature and calibrated using the signal of residual water (δ = 4.79) as an internal standard. The 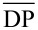 was calculated using the following equation:

[1] $$\bar{\text{DP}}=\frac{{\text{H}}_{1',1''}}{{\text{H}}_{1\alpha }+{\text{H}}_{1\beta }}+1\;,$$

where H_1’,1”_, H_1α_, and H_1β_ are the integrals of the corresponding protons (see the chemical structure of the cellulose oligomer in Figure 5).

For MALDI–TOF mass spectrometry, the purified product dispersions were mixed at a final concentration of 0.0033% (w/v) with 2,5-dihydroxybenzoic acid, trifluoroacetic acid, and MeCN at concentrations of 1.7 mg mL^−1^, 0.02 vol %, and 50 vol %, respectively. The mixtures were deposited on an AXIMA 384-well plate and dried under ambient conditions. An AXIMA-performance instrument (Shimadzu) equipped with a nitrogen laser (λ = 337 nm) and pulsed ion extraction was used at an accelerating potential of 20 kV in linear positive ion mode to obtain mass spectra. The spectra were calibrated using peptide standards (ProteoMass MALDI–MS Standard) at 757.3997 (bradykinin fragment 1–7), 1533.8582 Da (P_14_R), and 2465.1989 Da (ACTH fragment 18–39). The 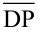 and the PSD of DP were calculated using the following equations:

[2] $$\bar{\text{DP}}=\frac{{\bar{\text{M}}}_{\text{n}}-18.0}{162}=\frac{\frac{\sum_{i}\left({\text{N}}_{i}{\text{M}}_{i}\right)}{\sum_{i}{\text{N}}_{i}}-18.0}{162}$$

[3] $$\text{PSD}\;\text{of}\;\text{DP}=\sqrt{\frac{\sum_{i}{\text{N}}_{i}{\left(i-\bar{\text{DP}}\right)}^{2}}{\sum_{i}{\text{N}}_{i}}}$$

where 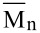 is the number average molecular weight, N*_i_* is the peak area of *i*-mer species, and M*_i_* is the molar mass of that species.

For XRD measurements, the lyophilized products were pressed into pellets using a hand press. A D8 DISCOVER instrument (Bruker) with Cu Kα radiation (λ = 1.542 Å) was operated under ambient conditions to obtain the transmission XRD patterns and transmitted X-ray intensities of the products using a two-dimensional (2D) detector and a scintillation counter, respectively. The 2D diffraction patterns were converted into 1D profiles in the 2θ range of 7–35°. The contribution of air scattering was subtracted from the 1D profiles based on the following equation:

[4] $$I_{\text{cor}}=I_{\text{obs}}-t\cdot I_{\text{blank}}$$

where *I*_cor_ is the corrected intensity, *I*_obs_ is the observed intensity, *t* is the X-ray transmittance through the sample, and *I*_blank_ is the intensity measured without any sample. The amorphous cellulose halo obtained previously [42] was fitted to the 1D profiles in 2θ ranges adequately selected from 15–20° for each profile. The χ_c_ was estimated according to the following equation:

[5] $$\chi_{\text{c}}=\frac{\int_{10{}^{\circ}}^{35{}^{\circ}}I_{\text{c}}\left(2\theta \right)\text{d}\left(2\theta \right)}{\int_{10{}^{\circ}}^{35{}^{\circ}}I\left(2\theta \right)\text{d}\left(2\theta \right)}\times 100$$

where *I*_c_(2θ) is the diffraction intensity from the crystalline phase, and *I*(2θ) is the intensity from both the crystalline and amorphous phases.

For ATR-FTIR absorption spectroscopy, the lyophilized products in a powdery state were used. The spectra were recorded on an FT/IR-4100 instrument (JASCO) at a cumulative measurement number of 100 and a resolution of 2.0 cm^−1^ under ambient conditions.

For SEM observations, the water solvent of the hydrogels after purification was exchanged stepwise with 10, 20, 30, 40, 50, 60, 70, 80 and 90 vol % EtOH, EtOH, EtOH/*tert*-butyl alcohol (1:1, v/v) and then *tert*-butyl alcohol by immersion. The obtained organogels were freeze-fractured using liquid nitrogen and a razor blade and then lyophilized. The obtained xerogels were mounted on substrates using Dotite and then coated with osmium. The fracture surface was observed by a field-emission scanning electron microscope (JSM-7500F, JEOL) at an accelerating voltage of 5 kV.

### Analysis of the secondary structure of CDP

The secondary structure of CDP was analyzed by CD spectroscopy. CDP was dissolved in a 8 mM phosphate buffer solution containing 10 vol % MeCN or EtOH at a concentration where the absorbance of CDP at 280 nm was 0.1. The CDP solutions were incubated at 60 °C for 6 and 72 h. The CD spectra of the samples were recorded on a J-725 instrument (JASCO) at a path length of 2 mm, a scan rate of 100 nm min^−1^, and a cumulative measurement number of 4.

## References

[1] Zou Q, Liu K, Abbas M, Yan X (2016). Adv Mater (Weinheim, Ger).

[2] Ariga K, Li J, Fei J, Ji Q, Hill J P (2016). Adv Mater (Weinheim, Ger).

[3] Malgras V, Ataee-Esfahani H, Wang H, Jiang B, Li C, Wu K C-W, Kim J H, Yamauchi Y (2016). Adv Mater (Weinheim, Ger).

[4] Komiyama M, Yoshimoto K, Sisido M, Ariga K (2017). Bull Chem Soc Jpn.

[5] Khan A H, Ghosh S, Pradhan B, Dalui A, Shrestha L K, Acharya S, Ariga K (2017). Bull Chem Soc Jpn.

[6] Sawada T (2017). Polym J.

[7] Komiyama M, Mori T, Ariga K (2018). Bull Chem Soc Jpn.

[8] Ariga K, Mori T, Shrestha L K (2018). Chem Rec.

[9] Sawada T, Serizawa T (2018). Bull Chem Soc Jpn.

[10] Azzaroni O, Ariga K (2019). Mol Syst Des Eng.

[11] Zhao L, Zou Q, Yan X (2019). Bull Chem Soc Jpn.

[12] Numajiri K, Kimura M, Kuzuya A, Komiyama M (2010). Chem Commun.

[13] Zhu G, Zheng J, Song E, Donovan M, Zhang K, Liu C, Tan W (2013). Proc Natl Acad Sci U S A.

[14] Zhu Z, Wu C, Liu H, Zou Y, Zhang X, Kang H, Yang C J, Tan W (2010). Angew Chem, Int Ed.

[15] Douglas S M, Bachelet I, Church G M (2012). Science.

[16] Liu M, Fu J, Hejesen C, Yang Y, Woodbury N W, Gothelf K, Liu Y, Yan H (2013). Nat Commun.

[17] Matsuda N, Shimizu T, Yamato M, Okano T (2007). Adv Mater (Weinheim, Ger).

[18] Souza G R, Molina J R, Raphael R M, Ozawa M G, Stark D J, Levin C S, Bronk L F, Ananta J S, Mandelin J, Georgescu M-M (2010). Nat Nanotechnol.

[19] Moon R J, Martini A, Nairn J, Simonsen J, Youngblood J (2011). Chem Soc Rev.

[20] Ifuku S, Saimoto H (2012). Nanoscale.

[21] Kadokawa J-i (2011). Chem Rev.

[22] Chang C, Zhang L (2011). Carbohydr Polym.

[23] Wang H, Gurau G, Rogers R D (2012). Chem Soc Rev.

[24] Habibi Y, Lucia L A, Rojas O J (2010). Chem Rev.

[25] Klemm D, Kramer F, Moritz S, Lindström T, Ankerfors M, Gray D, Dorris A (2011). Angew Chem, Int Ed.

[26] Muzzarelli R, Mehtedi M, Mattioli-Belmonte M (2014). Mar Drugs.

[27] Kobayashi S, Sakamoto J, Kimura S (2001). Prog Polym Sci.

[28] Shoda S-i, Uyama H, Kadokawa J-i, Kimura S, Kobayashi S (2016). Chem Rev.

[29] Kobayashi S, Kashiwa K, Kawasaki T, Shoda S (1991). J Am Chem Soc.

[30] Hiraishi M, Igarashi K, Kimura S, Wada M, Kitaoka M, Samejima M (2009). Carbohydr Res.

[31] Serizawa T, Kato M, Okura H, Sawada T, Wada M (2016). Polym J.

[32] Samain E, Lancelon-Pin C, Férigo F, Moreau V, Chanzy H, Heyraud A, Driguez H (1995). Carbohydr Res.

[33] Serizawa T, Fukaya Y, Sawada T (2017). Langmuir.

[34] Yataka Y, Sawada T, Serizawa T (2015). Chem Commun.

[35] Yataka Y, Sawada T, Serizawa T (2016). Langmuir.

[36] Nohara T, Sawada T, Tanaka H, Serizawa T (2016). Langmuir.

[37] Wang J, Niu J, Sawada T, Shao Z, Serizawa T (2017). Biomacromolecules.

[38] Adharis A, Petrović D M, Özdamar I, Woortman A J J, Loos K (2018). Carbohydr Polym.

[39] Nohara T, Sawada T, Tanaka H, Serizawa T (2017). J Biomater Sci, Polym Ed.

[40] Nohara T, Sawada T, Tanaka H, Serizawa T (2019). Bull Chem Soc Jpn.

[41] Lee J H, Brown R M, Kuga S, Shoda S, Kobayashi S (1994). Proc Natl Acad Sci U S A.

[42] Hata Y, Sawada T, Marubayashi H, Nojima S, Serizawa T (2019). Langmuir.

[43] Hata Y, Kojima T, Koizumi T, Okura H, Sakai T, Sawada T, Serizawa T (2017). ACS Macro Lett.

[44] Hata Y, Sawada T, Serizawa T (2017). Polym J.

[45] Hata Y, Sawada T, Sakai T, Serizawa T (2018). Biomacromolecules.

[46] Serizawa T, Fukaya Y, Sawada T (2018). Polym J.

[47] Cavallaro G, Lazzara G, Konnova S, Fakhrullin R, Lvov Y (2014). Green Mater.

[48] Tran A, Hamad W Y, MacLachlan M J (2018). ACS Appl Nano Mater.

[49] Lee W S, Choi J (2019). ACS Appl Mater Interfaces.

[50] Kurečič M, Mohan T, Virant N, Maver U, Stergar J, Gradišnik L, Kleinschek K S, Hribernik S (2019). RSC Adv.

[51] Nelson M L, O'Connor R T (1964). J Appl Polym Sci.

[52] Bittiger H, Husemann E (1972). J Polym Sci, Part B: Polym Lett.

[53] Buleon A, Chanzy H (1978). J Polym Sci, Polym Phys Ed.

[54] Buffiere J, Abad N, Ahvenainen P, Dou J, Cocero M J, Sixta H (2018). ACS Sustainable Chem Eng.

[55] Ellis R J (2001). Trends Biochem Sci.

[56] Nakano S-i, Miyoshi D, Sugimoto N (2014). Chem Rev.

[57] Hata Y, Sawada T, Serizawa T (2018). J Mater Chem B.

[58] Marcus Y (1993). Chem Soc Rev.

[59] Jessop P G, Jessop D A, Fu D, Phan L (2012). Green Chem.

[60] Kuroda K, Satria H, Miyamura K, Tsuge Y, Ninomiya K, Takahashi K (2017). J Am Chem Soc.

