# Correction: Biocatalytic oligomerization-induced self-assembly of crystalline cellulose oligomers into nanoribbon networks assisted by organic solvents

**DOI:** 10.3762/bjnano.11.27

**Published:** 2020-02-19

**Authors:** Yuuki Hata, Yuka Fukaya, Toshiki Sawada, Masahito Nishiura, Takeshi Serizawa

**Affiliations:** 1 Department of Chemical Science and Engineering, School of Materials and Chemical Technology, Tokyo Institute of Technology, 2-12-1 Ookayama, Meguro-ku, Tokyo 152-8550, Japan; 2 Precursory Research for Embryonic Science and Technology (PRESTO), Japan Science and Technology Agency (JST), 4-1-8 Honcho, Kawaguchi-shi, Saitama 332-0012, Japan; 3 DKS Co. Ltd., 5 Ogawaracho, Kisshoin, Minami-ku, Kyoto-shi, Kyoto 601-8391, Japan

**Keywords:** cellulose oligomer, gel, nanoarchitectonics, nanoribbon networks, oligomerization-induced self-assembly, organic solvent

The originally published Figure 4 contains wrong units on the y axes of both depicted CD spectra. The corrected Figure 4 is given below.

**Figure 1:**
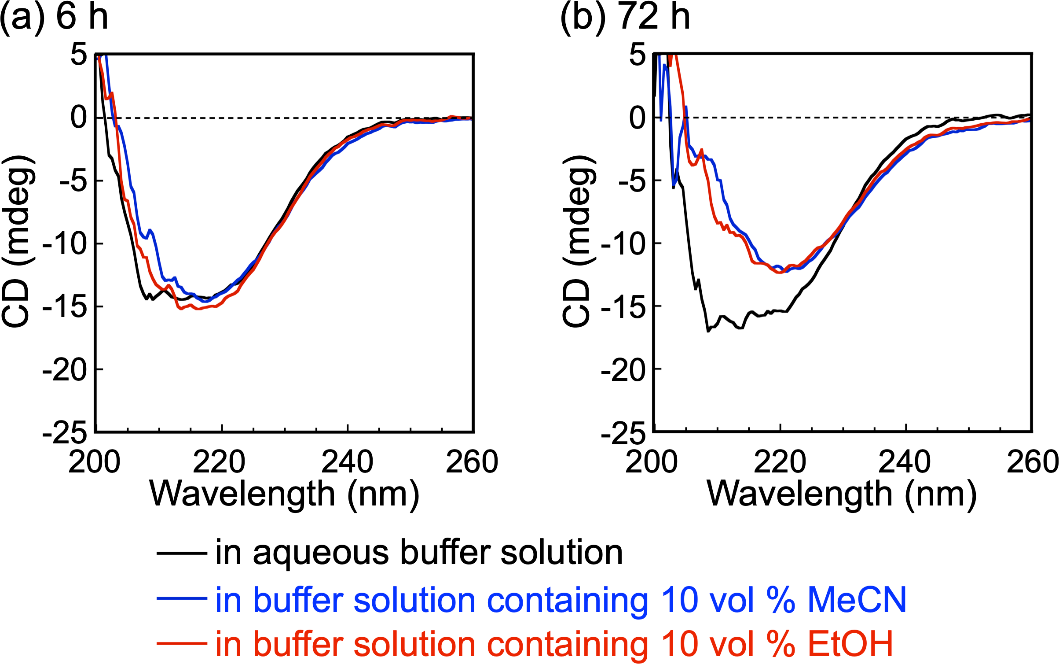
Corrected Figure 4 of the original publication.

